# The Design and Implementation of Adsorptive Removal of Cu(II) from Leachate Using ANFIS

**DOI:** 10.1155/2013/590267

**Published:** 2013-06-11

**Authors:** Nurdan Gamze Turan, Okan Ozgonenel

**Affiliations:** ^1^Department of Environmental Engineering, Engineering Faculty, Ondokuz Mayıs University, Samsun, Kurupelit 55139, Turkey; ^2^Department of Electric and Electronic Engineering, Engineering Faculty, Ondokuz Mayıs University, Samsun, Kurupelit 55139, Turkey

## Abstract

Clinoptilolite was investigated for the removal of Cu(II) ions from industrial leachate. Adaptive neural fuzzy interface system (ANFIS) was used for modeling the batch experimental system and predicting the optimal input values, that is, initial pH, adsorbent dosage, and contact time. Experiments were studied under laboratory batch and fixed bed conditions. The outcomes of suggested ANFIS modeling were then compared to a full factorial experimental design (2^3^), which was utilized to assess the effect of three factors on the adsorption of Cu(II) ions in aqueous leachate of industrial waste. It was observed that the optimized parameters are almost close to each other. The highest removal efficiency was found as about 93.65% at pH 6, adsorbent dosage 11.4 g/L, and contact time 33 min for batch conditions of 2^3^ experimental design and about 90.43% at pH 5, adsorbent dosage 15 g/L and contact time 35 min for batch conditions of ANFIS. The results show that clinoptilolite is an efficient sorbent and ANFIS, which is easy to implement and is able to model the batch experimental system.

## 1. Introduction

Industrial wastewaters, which have heavy metals, are an important source of environmental pollution. Pb, Cd, Cu, Hg, Cr, N, and Zn are the main trace elements that are the most harmful for public health and toxic priority pollutants. They commonly interfere with the beneficial use of wastewater for irrigation and industrial applications [1–4]. Moreover, heavy metals severely limit the beneficial use of water for domestic or industrial applications.

Removal of heavy metals can be accomplished by a variety of techniques. Conventional methods typically involve the use of processes such as coagulation, precipitation, ion-exchange, electrochemical methods, membrane processes, extraction, biosorption, and adsorption [5–7]. However, many of these methods can be marginally cost-effective or difficult to implement in developing countries. Among these methods, adsorption is currently considered to be very suitable for wastewater treatment because of its simplicity and cost-effectiveness [8–11]. 

Activated carbon is a widely used adsorbent for adsorption of metal ions [12, 13]. Even though it has a high adsorption capacity, surface area, and microporous structure, it is restricted to use due to its relatively high price, high operation costs, and problems with regeneration for the industrial scale applications. This led to a search directed to developing the low-cost and locally available adsorbent materials with the maximum adsorption capacity. 

Among natural and synthetic microporous materials, zeolites are characterized by high specific surface area and high cation exchange capacities. Because of many valuable properties such as sorption ability, catalytic, ion exchange, and atom/molecule trapping capabilities, zeolites are of great interest to material science [14–17].

Clinoptilolite is the most common and abundant high-siliceous zeolite. Clinoptilolite is crystalline, hydrated aluminosilicate of alkali and alkaline earth cations possessing an infinite, open three-dimensional structure [18]. The microporous crystalline structure of clinoptilolite is able to adsorb species that have diameters that fit through surface entry channels, while larger species are excluded, giving rise to molecular sieving properties that are exploited in a wide range of commercial applications [19, 20]. Clinoptilolite has particularly effective removal of lead [21–23], cadmium [24–26], thorium [27–29], zinc [30–32], manganese [33–36], and ammonium [37–39] from effluents. It has also found application in removal and purification of cesium and strontium radioisotopes [40, 41].

Clinoptilolites nowadays are mostly used in catalysis, in air enrichment, as filters in paper and rubber industry, in soil beneficiation, as animal feed supplements, and in water and wastewater treatment [42].

In this work, clinoptilolite has been investigated for the removal of Cu(II) ions from industrial leachate. Adaptive neural fuzzy interface system (ANFIS) is proposed to model the experimental system and predict the removal efficiency. The suggested technique is compared to 2^3^ full factorial experimental design based on ANOVA and *F*-tests. To the authors' knowledge, ANFIS is first used for adsorption studies.

## 2. Material and Method

### 2.1. Adsorbent

**Figure 1:**
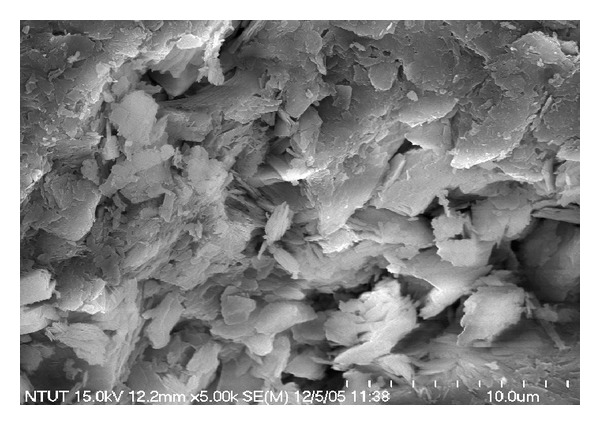
SEM microphotograph of clinoptilolite.

Clinoptilolite was obtained from Rota Mining Industry & Trade Co. Ltd., Manisa-Gördes, Turkey. For experimental studies, the mineral was washed with distilled water to remove any nonadhesive impurities and small particles and then dried at 70°C for 24 h to remove moisture. The samples were sieved through 0.6 mm sieve and used as such without any treatments. Finally, clinoptilolite samples were stored in separate vacuum desiccators until required. The chemical composition of the mineral was evaluated by using X-ray fluorescence techniques (Spectro-Xepos). The chemical composition of the mineral is presented in Table 1. The mineral contained significant levels of SiO_2_ (71.00%) and Al_2_O_3_ (11.80%), while the contents of other metal oxides were less than 10%. The SEM image of the waste is shown in Figure 1. Clinoptilolite samples appear as corn flake like crystals with fluffy appearance revealing its extremely fine platy structure (Figure 1).

### 2.2. Industrial Waste

The copper flotation waste as industrial waste was used for this study. The wastes were obtained from the ETI Copper Works in Samsun, Turkey. The chemical composition of the waste was evaluated by using X-ray fluorescence techniques (Spectro-Xepos). The chemical analysis of the waste was given in Table 1. The copper flotation waste contained significant levels of Fe_2_O_3_ (67.68%) and SiO_2_ (24.87%) (Table 1).

### 2.3. Leaching Tests

ASTM test methods were used to evaluate the leaching and pollution potentials of pollutants in the waste in this study. Standard 1 : 4 (w/w extractant to sample) mixtures were performed using the deionized water in a Teflon bottle. Bottles were shaken for 48 h at 25°C on an end-over-end rotary shaker rotating at 200 rpm. Leachate was filtered (0.22 *μ*m openings) and used as leaching solution in the adsorption experiments [43].

### 2.4. Adsorption Procedure

The adsorption of Cu(II) from industrial leachate onto clinoptilolite was performed using the batch equilibrium technique. All batch experiments were conducted with adsorbent samples with 100 mL. Erlenmeyer flasks closed with glass stoppers in a thermostated shaking water bath to elucidate the optimum conditions of pH, adsorbent dosage, and contact time. 

The input factors such as pH, adsorbent dosage, and contact time are changed as 3 and 6, 5 and 20 g L^−1^, 20 and 60 min, respectively. These are minimum and maximum values and simply chosen as levels of the related factors. A total of 8 experiments were done and the batch experiments were duplicated to increase the reliability of the experimental system.

### 2.5. ANFIS Model

The overall experimental procedure is modeled by using ANFIS, which is the abbreviated of adaptive neurofuzzy interface system. Some authors previously used artificial neural networks (ANNs) to model the adsorption system and predict the removal efficiency [44–50]. ANN is a system of data processing based on the structure of a biological neural system. The prediction with ANN is made by learning of the experimentally generated data or using validated models. In classical ANN structure, the prediction can be performed after several iterations (computer runs) by a number neurons in layers. 

ANFIS is similar to fuzzy interface system, which has been first introduced by Zadeh [51], by using a backpropagation trying to minimize the error. Therefore, the performance of ANFIS is like both ANN and fuzzy logic [52]. In ANFIS, the input passes through the input layer (by input membership function) and the output is seen in output layer (by input membership function). 

In this paper, a new approach based on ANFIS is presented to predict the adsorption efficiency of Cu(II) from industrial leachate. 

**Figure 2:**
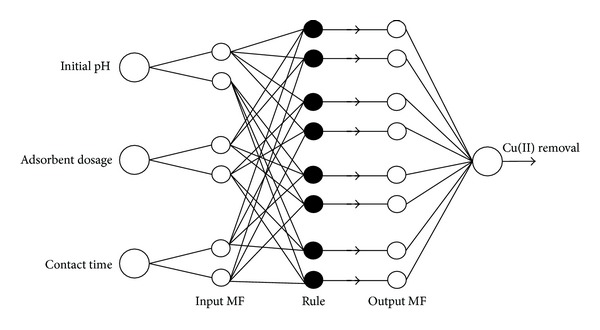
Proposed ANFIS structure for Cu(II) removal system.

Figure 2 shows the proposed ANFIS structure for Cu(II) removal system. A typical ANFIS structure consists of 5 layers.

In Figure 1, the first layer is the input layer. Initial pH, adsorbent dosage, and temperature are the inputs of the experimental procedure. The whole experimental design used in ANFIS and statistical calculations are given in Table 2. 

The layers of *inputmf* and *outputmf* are the fuzzy parts of ANFIS and are mathematically incorporated in the form of membership functions (MFs). An MF, *f*(*x*; *a*, *b*, *c*), can be any continuous and piecewise differentiable function that transforms the input/output value into a membership degree (a value between 0 and 1). The most widely applied MF is the generalized bell (gbell). However, many MFs are tried and their performances on the Cu(II) removal efficiency are compared.  Table 3 shows the MFs and their associated mathematical representations.

The output layer is the summation of the net outputs and gives the Cu(II) removal efficiency. Each input has two MFs for ANFIS learning. Iteration number is set to 1 for this particular example. The minimum error according to training procedure is obtained by using pi-shaped MF (7.71*E* − 6). Therefore, the effects of pH, adsorbent dosage, and contact time are investigated by pi-shape MF.

### 2.6. Effect of pH, Adsorbent Dosage, and Contact Time

To investigate the main and interaction effects of each input, each input is changed in a range of *low*_*value* − 1 ≤ *input* ≤ *high*_*value* + 1, while the other inputs are kept as low and high values. Figure 2 shows the effect of initial pH. The range of pH, *A*, is set to 2–7 while *B* and *C* are changed as low-high values in experimental design.

**Figure 3:**
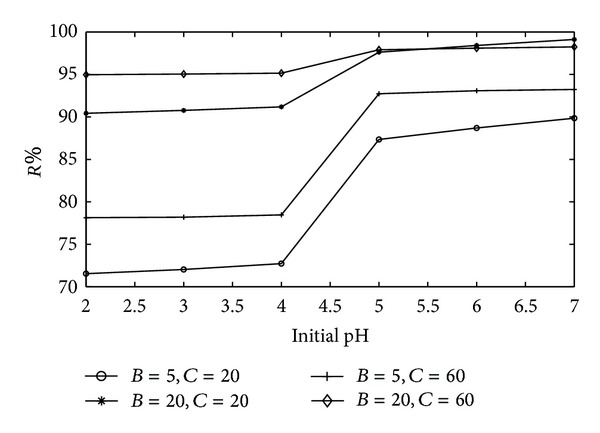
The effect of initial pH on Cu(II) removal.

The values of removal efficiencies do not change significantly after the pH value of 5 (Figure 3). Therefore, pH value of 5 is chosen as optimal value. Average value of removal efficiency is defined in (1) for pH variation:

(1) $$\begin{array}{c} \text{A}{\text{V}}_{\text{pH}}=\frac{1}{N}\overset{N}{\underset{k=1}{\sum }}\left(\text{Curve}1_{k}+\text{Curve}2_{k}+\text{Curve}3_{k}+\text{Curve}4_{k}\right). \end{array}$$

**Figure 4:**
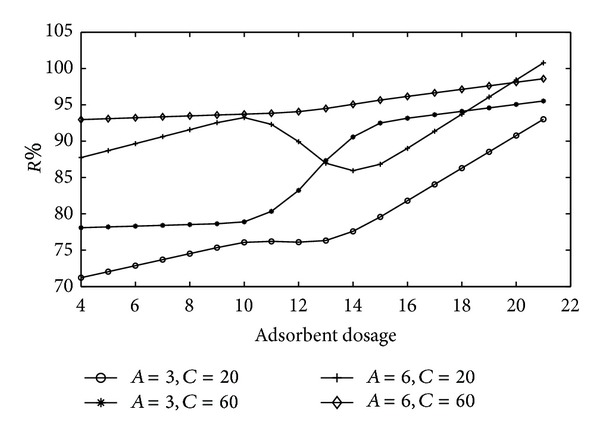
The effect of adsorbent dosage (g/L) on Cu(II) removal.

Figure 4 shows the effect of adsorbent dosage. The range of adsorbent dosage,  *B*, is set to 4–21 while *A* and *C* are changed as low-high values in experimental design. 

The values of removal efficiencies do not change significantly after the adsorbent dosage of 15 g/L. Figure 4 also shows the strong relationship between adsorbent dosage and initial pH. 

Average value of removal efficiency is defined in (2) for adsorbent dosage variation:

(2) AVadsorbent_dosage =1N∑k=1N(Curve1k+Curve2k+Curve3k+Curve4k).

**Figure 5:**
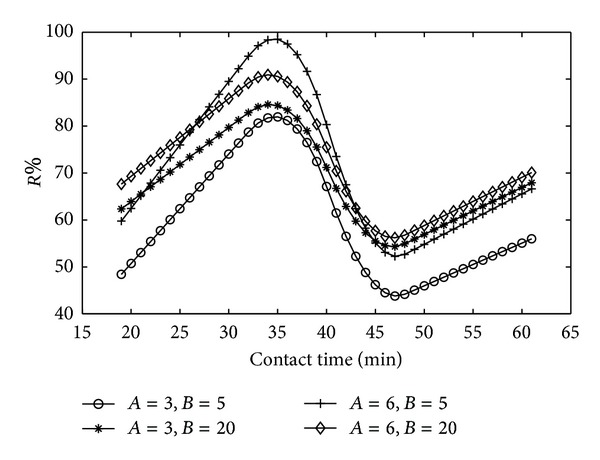
The effect of contact time (min) on Cu(II) removal.

Figure 5 shows the effect of contact time. The range of contact time,  *B*, is set to 19–61 while *A* and *B* are changed as low-high values in experimental design. 

The maximum removal efficiencies are obtained at 35 min of contact time. Therefore, it is selected as optimum value of contact time. Similarly, average value of removal efficiency is defined in (3) for contact time variation:

(3) AVcontact_time =1N∑k=1N(Curve1k+Curve2k+Curve3k+Curve4k).

The overall average value of Cu(II) removal is calculated in (4):

(4) $$\begin{array}{c} \text{A}{\text{V}}_{\text{Cu}(\text{II})}=\frac{1}{3}\left(\text{A}{\text{V}}_{\text{pH}}+{\text{AV}}_{\text{adsorbent}\_\text{dosage}}+\text{A}{\text{V}}_{{\text{contact}\_\text{time}}_{}}\right). \end{array}$$

According to (4), the overall average value of Cu(II) removal is calculated as 90.43% at the optimal values of pH, adsorbent dosage, and contact time.

### 2.7. Validating the Results with Statistical Analysis

Statistical analysis is based on 2^3^ full factor experiments. In order to decide the accuracy of the models among various models that are linear, quadratic, or cubic, to display Cu(II) removal by clinoptilolite, two different tests known as sequential model sum of squares and model summary statistics were carried out in the suggested work.

ANOVA is a statistical technique that subdivides the total variation in a set of data into element items relating to specific sources of variation for the purpose of testing hypotheses on the parameters of the model. The statistical significance of the ratio of mean square variation due to regression and mean square residual error was tested using ANOVA method. The ANOVA for the fitted equations is shown in Table 4 showing the performance of the full factorial design. The ANOVA results showed that the equations adequately represented the actual relationship between the response and significant variables. According to Table 4, the Model *F*-value of 105.72 implies that the model is significant and values of “Prob > *F*” less than 0.05 indicate model terms are significant. Therefore, in this case *A* and *B* are the significant model terms. 

The “predicted *R*-squared” of 0.8993 is in reasonable agreement with the “adjusted *R*-squared” of 0.9890. “adequate precision” measures the signal-to-noise ratio. A ratio greater than 4 is desirable. The calculated ratio of 28.69 indicates an adequate signal. As a result, this model can be used to navigate the design space.

**Figure 6:**
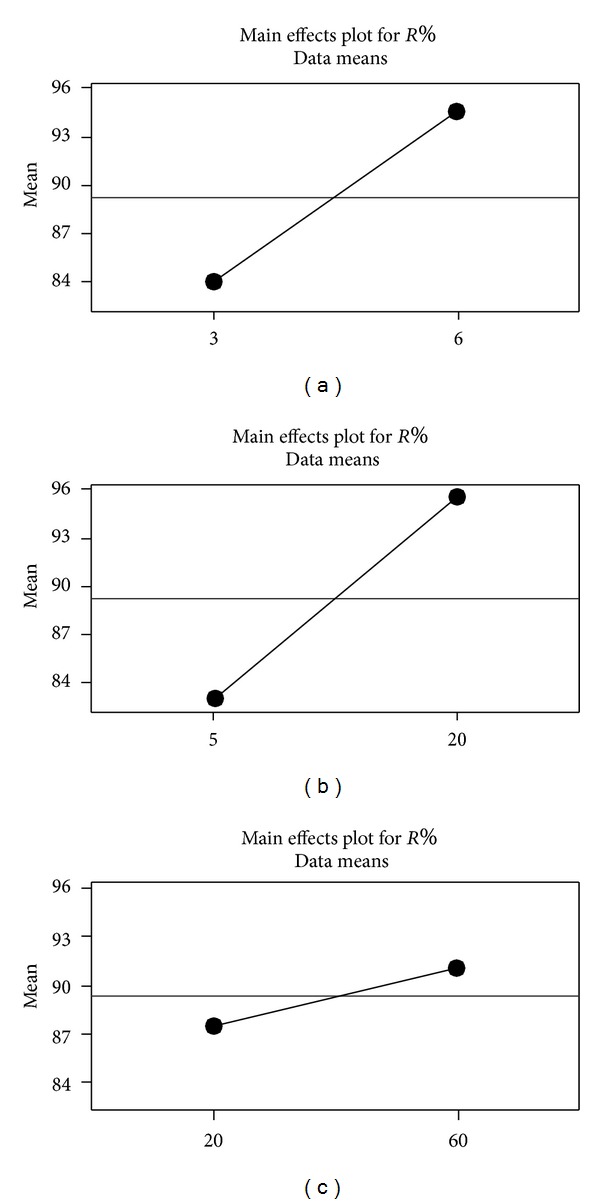
Main effects of analyzed factors.

**Figure 7:**
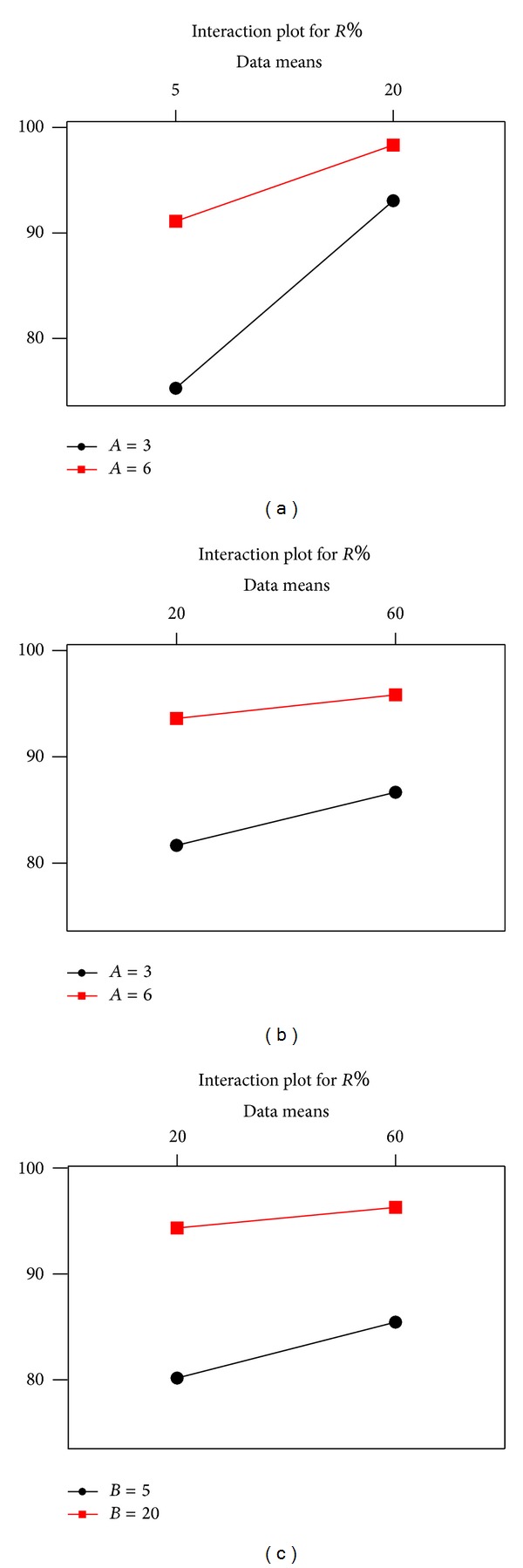
Interaction effects of analyzed factors.

Figures 6 and 7 show the main and interaction effects for analyzed factors. As seen from Figures 5 and 6, the main factors *A* and *B* are statistically significant terms and there is also a significant relationship between the terms *A* and *B*. The effect of contact time,  *C*, on removal efficiency has statistically less importance than the effects of *A* and  *B*. 

Values greater than 0.1 indicate the model terms are not significant (Table 4). Final regression equation in terms of coded factors is given in (5):

(5) $$\begin{array}{c} R\%=89.3+5.28A+6.29B. \end{array}$$

**Figure 8:**
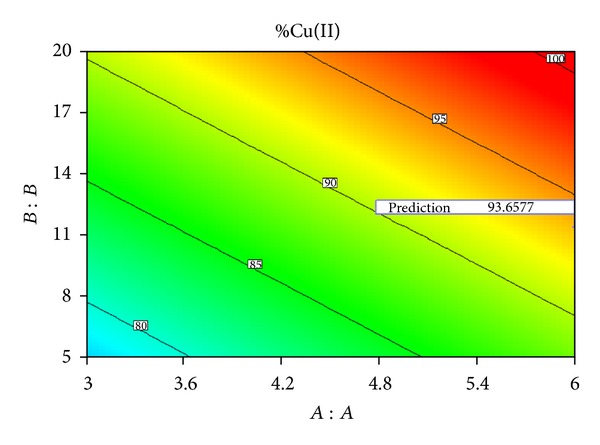
Prediction of removal efficiency with optimized parameters.

According to optimization procedure, the optimized input variables are calculated as initial pH of 6, adsorbent dosage of 11.4 g/L, and contact time of 33 min with a removal capacity of 93.65%.  Figure 8 shows the optimization cube with a constant contact time of 33 min.  Table 5 shows the comparison of ANFIS and statistical analysis on optimized factors and predicted removal efficiencies.

Table 5 proves that the results are very close to each other. The suggested ANFIS model, which is easy to apply and a fast approach, proposes an alternative way to model adsorption studies.

## 3. Conclusion

Clinoptilolite was investigated for the removal of Cu(II) ions from industrial leachate and found to have more affinity for copper among the studied metals. In conclusion, the series of the real-time tests performed in this work shows that

- the removal of Cu(II) ions with clinoptilolite holds great potential for simple solution,
- an ANFIS model with three inputs and one output is proposed for modeling the dynamics of the adsorption process. The proposed ANFIS model does not require many parameters and complex calculations, which need to be predicted by time-consuming and expensive experiments,
- local maxima values of the performance graphics based on initial pH, adsorbent dosage, and contact time are regarded as optimal values in proposed ANFIS model,
- the interactions between the input factors and removal efficiency are also analyzed by 2^3^ full factorial experimental design followed by ANOVA and *F*-test and the results are compared to ANFIS model. According to the comparison, the values of optimum input parameters and removal efficiency are close to each other and in an acceptable error limit,
- higher-order experimental designs can easily be modeled by an ANFIS model and very accurate results can be obtained.

## Figures and Tables

**Table 1:** Chemical analysis of materials.

Components	%w/w	
	Clinoptilolite	Industrial waste
Na_2_O	0.40	—
MgO	1.40	0.36
Al_2_O_3_	11.80	0.92
SiO_2_	71.00	24.87
CaO	3.40	0.69
TiO_2_	0.10	0.08
K_2_O	2.40	0.48
F_2_O_3_	1.70	67.68
ZnO	—	2.78
CuO	—	0.98
PbO	—	0.21
MnO	—	0.12
CoO	—	0.21
SO_3_	0.12	2.18

NZ: natural zeolite, B: bentonite, FW: flotation waste.

**Table 2:** Experimental design (2^3^).

Initial pH, *A *	Adsorbent dosage (g/L), *B *	Contact time (min), *C *	%*R*
3	5	20	72.04
3	5	60	78.20
3	20	20	90.77
3	20	60	95.04
6	5	20	88.70
6	5	60	93.09
6	20	20	98.42
6	20	60	98.1

%*R*: removal efficiency of Cu(II) ions.

**Table 3:** The most common MFs and their associated expressions.

Triangular MF, trimf	$f(x;a,b,c)=\left\{\begin{array}{cc} 0, & x\leq a\\ \frac{x-a}{b-a}, & a\leq x\leq b\\ \frac{c-x}{c-b}, & b\leq x\leq c\\ 0, & c\leq x \end{array}\right\}$
Trapezoidal MF, trapmf	$f(x;a,b,c,d)=\left\{\begin{array}{cc} 0, & x\leq a\\ \frac{x-a}{b-a} & a\leq x\leq b\\ 1, & b\leq x\leq c\\ \frac{d-x}{d-c} & c\leq x\leq d\\ 0, & d\leq x \end{array}\right\}$
Generalized bell-shaped MF	$f(x;a,b,c)=\frac{1}{1+{\left(\left(x-c\right)/a\right)}^{2b}}$
Gaussian curve MF	*f*(*x*; σ, *c*) = *e* ^−(*x* − *c*)^2^/2σ^2^^
pi-shape MF	$f(x;a,b,c,d)=\left\{\begin{array}{cc} 0, & x\leq a\\ 2{\left(\frac{x-a}{b-a}\right)}^{2}, & a\leq x\leq \frac{a+b}{2}\\ 1-2{\left(\frac{x-b}{b-a}\right)}^{2}, & \frac{a+b}{2}\leq x\leq b\\ 1-2{\left(\frac{x-c}{d-c}\right)}^{2}, & c\leq x\leq \frac{c+d}{2}\\ 2{\left(\frac{x-d}{d-c}\right)}^{2} & \frac{c+d}{2}\leq x\leq d\\ 0, & x\geq d \end{array}\right\}$
MF composed of the difference between two sigmoidal MFs, dsigmf	$f(x;a,c)=\frac{1}{1+e^{-a(x-c)}}$
Product of the two sigmoid MF, psigmf	$f(x;a,c)=\frac{1}{1+e^{-a(x-c)}}$

**Table 4:** ANOVA for 2^3^ full factor experimental design.

Source	Sum of squares	d.f.	Mean square	*F* value	*P* value prop > *F *	Remark
Model	630.57	6	105.1	105.72	0.0743	
*A *	223.24	1	223.24	224.57	0.0424	Significant
*B *	316.26	1	316.26	318.15	0.0357	Significant
*C *	26.28	1	26.28	26.44	0.1223	
*AB *	54.291	1	54.291	54.61	0.0586	
*AC *	5.06	1	5.06	5.09	0.2657	
*BC *	5.44	1	5.44	5.48	0.2571	
Residual	0.99	1	0.99			
Corr. total	631.56	7				

*A*: initial pH; *B*: adsorbent dosage; *C*: contact time.

**Table 5:** Optimized factors and predicted removal efficiencies by ANFIS and 2^3^ full factorial design.

Source	*A *	*B* g/L	*C* min	*R*%
2^3^ full factorial design	6	11.4	33	93.65
ANFIS	5	15	35	90.43

*A*: initial pH; *B*: adsorbent dosage; *C*: contact time.
